# Impact of Physical Activity Intervention Programs on Self-Efficacy in Youths: A Systematic Review

**DOI:** 10.1155/2013/586497

**Published:** 2013-02-07

**Authors:** Rosa Cataldo, Janice John, Latha Chandran, Susmita Pati, A. Laurie W. Shroyer

**Affiliations:** ^1^Department of Pediatrics, Long Island Children's Hospital, Stony Brook University Medical Center, 101 Nicolls Road, HSC Level T11, Room 020, Stony Brook, NY 11794-8111, USA; ^2^Undergraduate Medical Education, Stony Brook University Medical Center, Stony Brook, NY 11794-8111, USA; ^3^Departments of Preventive Medicine and Surgery, Stony Brook University Medical Center, Stony Brook, NY 11794-8111, USA

## Abstract

Lack of physical activity has contributed to the nation's childhood obesity crisis, but the impact of physical activity on self-efficacy as a mediator of behavior change has not been examined. This systematic review (SR) describes the published evidence related to the impact of physical activity intervention programs on self-efficacy among youths. From January 2000 to June 2011, the Preferred Reporting Items for Systematic Reviews and Meta-Analyses (PRISMA) standards were used to identify publications from PubMed, PsychInfo, Web of Knowledge, and the Cochran Database of Systematic Reviews. The Cochrane Population, Intervention, Control, Outcome, Study Design (PICOS) approach guided this SR articles selection and evaluation process. Of the 102 publications screened, 10 original studies matched the SR inclusion criteria. The types of physical activity interventions and self-efficacy assessments for these 10 studies were diverse. Of the 10 included articles, 6 articles identified an improvement in post-self-efficacy assessments compared to baseline and 4 showed no effect. In conclusion, physical activity intervention programs may improve self-efficacy in youths. A standardized approach to classify and measure self-efficacy is required. Further research is needed to quantify the association of self-efficacy ratings after completing physical activity interventions with objective health improvements, such as weight loss.

## 1. Introduction

The prevalence of childhood obesity has increased dramatically in the United States (US) the last 20 years. After age gender-based adjustments, more than 30% of US children and adolescents surveyed in 2007-2008 were overweight with a body mass index (BMI) greater than the 85th percentile [1]. Young Americans suffer increasing morbidity from chronic diseases associated with obesity before reaching adulthood [2]. Although the etiology of obesity is multifactorial, weight loss can be achieved by diet and activity behavior modification [3]. In order to sustain weight loss, health behavior modifications must become lifestyle changes. 

Self-efficacy, a construct of Albert Bandura's social cognitive theory (SCT), is defined as the belief in one's own ability to achieve actions necessary to produce a desired effect [4]. It relates to an individual's confidence in achieving and maintaining behavioral change. Hence, it is reasonable to anticipate that physical activity intervention programs may benefit from incorporating SCT modifications and to evaluate self-efficacy as a component in determining behavioral change. Various types of self-efficacy as a mediator of behavioral change toward physical activity interventions have been described in the literature [5]. 

Self-efficacy has been described as a predictor of physical activity intervention outcomes rather than an independent outcome. Authors have concluded that self-efficacy served as a covariate, which impacted other psychosocial factors and indirectly influenced the success of an intervention [6]. Several original articles as well as reviews have supported the model that self-efficacy functioned as a potential mediator, within various types of physical activity programs in children [7–10].

Although improved self-efficacy has been associated with the compliance of health behavior modification interventions, few studies indicate that the intervention may influence self-efficacy. The directionality of the interaction between physical activity interventions and self-efficacy has not been clearly established. One review did report the effect of physical activity intervention outcomes on self-efficacy as a potential mediator of behavioral change in 4–12 year-old children from 1985 to 2006 [11]. While useful, the age parameters limited the literature selection in the prior review. Additionally, the literature has not been revised to date. The potential for physical activity programs to facilitate self-efficacy and promote health behavior change remains: self-efficacy may be the transformational “missing link” to innovatively address the growing obesity crisis.

This SR primary objective was to determine if physical activity related programs were associated with improved self-efficacy in children and adolescents (5–18 years old). Given the potential to impact long-term health for children and adolescents, a secondary objective was to evaluate the effect of physical activity self-efficacy ratings upon weight reduction goals. 

## 2. Methods

**Figure 1:**
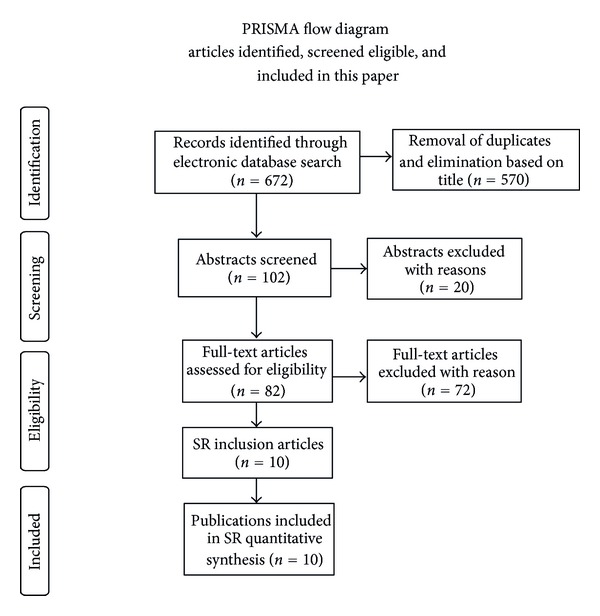


A protocol using the PRISMA standards was completed prior to initiating the literature search. Database searches were performed using PubMed, Web of Knowledge, PsychInfo, and the Cochran Database of Systematic Reviews (January 2000–June 2011). All appropriate titles and abstracts were reviewed per study inclusion/exclusion criteria. Due to a judicious electronic search, nonelectronic sources of literature were not considered. A detailed search strategy including search terms and limitations is listed in Appendix. Only those articles written in English were appraised. The Cochrane PICOS approach was applied to select the articles included (Figure 1).

To meet the SR inclusion criteria, each publication must have included a subject population of children and adolescents (5–18 years). In evaluating these publications, a special emphasis was placed stratifying the data abstracted for the subpopulations of overweight youths. Publications were excluded if participants had a medical illness. 

The articles reviewed were required to include a structured physical activity program lasting 4 weeks or more. School and community-based physical activity incorporating individual or group activities were included. Types of physical activity considered for this paper included: cardiovascular activity, resistance training, or modifications of physical education classes. Programs that included exercise exclusively or in conjunction with nutrition and psychosocial factors were considered. When provided, the level of physical activity was classified as moderate, moderate to vigorous, or vigorous preferably using metabolic equivalents (METs) rating. Publications were not required to document energy cost of physical activity, as this would have significantly limited the paper. Exclusively web-based programs were eliminated from the paper due to entirely self-reported assessments of physical activity.

All publications reviewed were required to include either a comparison or a control group. The type of comparison group permitted was liberal. The study participants may have been compared to subjects of another separate program, which was not coordinated contemporaneously. Comparisons may have also included groups that underwent an assessment period where there was no concurrent intervention, but then served as future intervention groups. 

The outcome measures for this SR included any self-reported physical activity self-efficacy and were required to be stated within the hypothesis or objectives of the study. Other motivational variables may have been included within the articles but were not addressed in this SR. All included publications must have identified statistically significant improvements (designated a priori), of self-efficacy after completion of the intervention. Studies that assessed objective “success” measures related to body weight, body mass index or body composition were accepted but not required as part of the SR inclusion criteria.

To assure the quality of the study findings reported, the Oxford Centre for Evidence-Based Medicine (Levels of Evidence) 5-level hierarchical tool was used. Only studies with Oxford Centre for Evidence-Based Medicine levels of 3 or higher were reported [12]. Hence, all articles used either a randomized control trial approach, a quasiexperimental, or observational study design. The experimental studies were further appraised with an assessment developed by Jadad et al. to grade clinical trials [13]. A slight modification of the Jadad scoring approach was used to assess the quasiexperimental studies. For the articles meeting inclusion criteria, Table 1 identifies each reviewed study's characteristics including all PICOS components. 

 As an assessment of inter-rater reliability, two authors independently reviewed each publication using a standardized data capture form with definitions to evaluate if all SR article inclusion/exclusion criteria were met as well as to appraise the quality. 

## 3. Results

### 3.1. Screening and Identification

Search strategies of the initial abstract screening identified 102 publications (Figure 1). Interventional, observational studies, reviews, and a meta-analysis were represented within the screened articles. Of the 102 screened publications, 10 original studies (11 articles) matched the final PICOS SR inclusion criteria [14–23]. One group of investigators used the same study population, study design, and data analysis methods to measure self-efficacy in two different publications. The two articles differed in addressing other psychosocial variables as well as how the multiple potential predictors for outcomes may have affected one another. These two articles were accounted for only once in this SR results and population size data to avoid overweighting of the findings [17, 24].

Seven interventional studies met all the inclusion criteria from the 20 screened interventional articles, yielding a 35% inclusion rate [17–24]. Three observational studies met all of the inclusion criteria from 70 screened articles, yielding a 4% inclusion rate [14–16].

 A comprehensive list of the eliminated articles with rationale for exclusion and references is represented in Table 5. Based upon the listed exclusion criteria: 15 studies did not fit the criteria for study design (16%), 39 studies used self-efficacy as a predictor of physical activity (42%), 26 articles (28%) were omitted based on population age criteria, and 61 articles (65%) did not fulfill the physical activity intervention criteria. 

### 3.2. PICOS Characteristics and Statistical Approaches

#### 3.2.1. Population

 A total of 5229 school age participants were enrolled across 3 different types of settings. Important race and gender-based variations were noted. Two studies included female participants only [17, 18, 24], 3 studies were comprised exclusively of African American participants [15, 16, 23], 2 studies included a majority of African American participants [14, 22], and one study focused upon an underserved Native American Indian population [20]. Age differences also varied across the studies. Three of the studies focused solely on children (<12 years of age) [15, 20, 21], 3 studies recruited only adolescents (12 to 18 years) [17–19, 24], and 4 studies focused on both children and adolescents [14, 16, 22, 23].

#### 3.2.2. Intervention

 All of the included publications used a multidisciplinary physical activity approach. Six of the studies employed a school-based setting [17–22, 24], one incorporated an after-school setting [23], and 3 were held at a Young Men's Christian Association (YMCA) location [14–16].

The key dimensions of the physical activity intervention components such as duration, intensity, and session length were diverse. The time frame of the programs varied from 8 weeks to 3 years. Six studies reported an intermediate duration (≥12 weeks up to 6 months) [14–16, 18, 19, 23], and 4 studies reported a longer program (≥1 year up to 3 years) [17, 20–22, 24]. The amount of sessions per week was as follows: 1 article reported intervention 2 days per week [19], 5 articles reported physical activity at least three days per week [14–16, 22, 23], 1 article reported five days per week [18], and 3 articles did not mention how often the physical activity sessions occurred [17, 20, 21, 24]. Across these publications, the length of each physical activity session ranged from 20 minutes up to 45 minutes. The assessment of metabolic equivalents (METS) was not captured and/or reported uniformly across all studies. Of the included studies: 5 reported measurements of moderate-to-vigorous intensity [14–16, 22, 23], one measured peak oxygen uptake (VO_2_ max) [18], one measured muscular strength [19], and 3 studies did not report intensity per session [17, 20, 21, 24].

#### 3.2.3. Comparisons

 Four studies were randomized control trials (RCTs), such that the control group was randomly assigned for the analytical comparisons performed [17, 19–21, 24]. The quasiexperimental (2 middle schools [22, 23], one high school [18]) studies designated partnerships with other schools to identify grade level matched comparison groups [18, 22, 23]. One of these studies also incorporated a more refined high school student matched comparison group based upon age, gender, and race [22].

For the three cohort studies, the comparisons to the physical activity related intervention programs varied. All 3 of the physical activity related interventions studied occurred in a local YMCA. In one of these studies, the comparison was a school-based physical education class [16]. The individual students were not randomized, and the students received their school's assigned intramural sports program. For the other two cohort studies, the comparison groups included: YMCA participants that were waitlisted to enroll in a future physical activity related program [15] and general YMCA participants that were not affiliated with any physical activity related intervention [14]. 

#### 3.2.4. Outcomes

 The “gold standard” description of self-efficacy is for perceived self-efficacy. In perceived self-efficacy, individuals have the belief that they are capable of functioning at a certain level of performance [4]. Several different categories of self-efficacy, as it applies to health-related behaviors, have been cited in the literature [25]. With the exception of the work of Annesi et al. [14–16] and Wilson et al. [22, 23], the self-efficacy assessments were not consistent across the publications included in this SR (Table 2). The lack of homogeneity of the self-efficacy surveys administered made it difficult to make in-depth comparisons to summarize the findings across studies. 

The approaches to identifying statistical associations within the included articles were diverse. For each article reviewed, a *P* value of ≤0.05 was used to identify if statistically significant associations were reported. The statistical methods used include (1) descriptive assessments of central tendency and variability [22]; (2) univariate comparison using *t*-tests, ANOVA, and chi squared tests [23]; (3) multivariable assessments using latent variable structural equation model [17, 24], structural equation model [19], simplistic regression analysis [18], and more complex mixed model analysis [14, 15, 20].

#### 3.2.5. Study Designs

 Of the included articles identified for an in-depth review, the array of study designs included 4 RCTs [17, 19–21, 24] and 3 quasiexperimental [18, 22, 23] and 3 cohort studies [14–16]. Incorporating a range of study designs within the parameters of the methods (Oxford level ≥ 3) was essential to strengthen the finding of this SR.

Per the preestablished SR protocol, the first SR project objective was to evaluate the association of physical activity intervention programs with improvements in self-efficacy. Of the 10 included studies six (60%) found an association with improved self-efficacy after intervention [14–16, 18, 20, 23]. One of these studies reported the association for females but not for males [20]. 

The second SR study objective was to identify self-efficacy ratings with associated achievement of weight reduction following completion of physical activity related intervention. Although most of the studies collected baseline body weight or BMI, none of them evaluated the association of self-efficacy ratings with achievement of weight reduction (Table 3).

Two of the coauthors (R. Cataldo, J. John) analyzed each of the 102 publications. Independent assessments meeting each study's preestablished inclusion/exclusion criteria, reason for exclusion, the Oxford level of evidence grade, and the Jadad quality assessment score were recorded by both reviewers. The following concordance was observed: (1) for inclusion/exclusion decisions, there was 100% final inter-rater agreement as well as a high concordance (96% agreement) related to the reason for exclusion; (2) for the Oxford grades (grades 1, 2, or 3), there was a 90% agreement for ratings assigned independently; (3) of the 5 articles where Jadad ratings were assessed, there was 1 article of disagreement (80% agreement). For the articles where initial disagreement was identified, a coauthor team consensus was reached for the data reported in Table 4. 

## 4. Discussion

Based on this SR, there is moderately strong evidence to suggest that physical activity intervention programs may improve self-efficacy. Given the expansion of childhood obesity in America, the question remains: how do we elicit self-efficacy for health behavior change? This paper suggests that exercise combined with a multidisciplinary approach may positively influence self-efficacy assessments in children and adolescents. In theory, once self-efficacy is obtained for a specific behavior, there is a potential for continuation of the desired health outcome. 

In efforts to campaign for a resolution of the obesity crisis, a secondary aim of this SR was to assess whether the physical activity related programs demonstrated weight reduction or weight maintenance. Of the included studies, none evaluated the association of improved self-efficacy with changes in body weight or BMI from before to after program. Due to the variable characteristics of each study design, we were unable to extrapolate any association between the improved self-efficacy ratings with objective changes in body weight or BMI. 

Self-efficacy surveys have evolved to correspond to the distinct theme for which they are necessary. Of the 10 studies evaluated, only two of the authors repeatedly used the same self-efficacy assessment in their respective studies. As an important limitation, the lack of continuity of the self-efficacy surveys made it difficult to generalize the results. 

 There were several limitations that may present as potential biases within the cohort studies. The types of physical activity interventions were diverse (levels of activity, types of activity, differences within the instructor's level of education, and varied locations). Additionally, participants' general interest in an extramural activity (intervention group) compared to a required physical education class may have also influenced the outcome. Despite these limitations, using exclusively RCT study designs for this particular topic would have significantly limited this study's findings. Given the heterogeneity of the studies reviewed, it was difficult to make a definitive judgment of the outcomes. Although many studies indicated the importance of physical activity and nutrition education for obesity, they did not focus solely on obese children. Therefore, participant selection bias may have been a potential confounder. Although many initially reviewed articles included baseline and postphysical activity program self-efficacy assessments, few articles appraised whether the physical activity curriculum details (e.g., dose of physical activity, duration of physical activity, and/or frequency of physical activity) were associated with an improved self-efficacy. The most challenging bias was the lack of a uniform, standardized definition of self-efficacy, as well as an inconsistent approach to measuring theory. The heterogeneous characterization of the term self-efficacy (psychosocial variable, self-efficacy, perceived barriers, internal verses external barriers) may have affected and limited the search criteria for articles in this SR.

The findings of this SR suggest that there is moderately strong evidence that physical activity related programs improve self-efficacy in youths. However, based on this paper, there is insufficient evidence about the effect of physical activity related programs on weight status. Sustaining the benefits of health behavior programs, whether improved self-efficacy or objective indicators (body weight), may be a key determinant to long-term health outcomes. Factors that influence persistence of positive behavioral changes over time need further elucidation. 

 Further research appears warranted to clarify the relationship between physical activity programs with changes in self-efficacy and weight loss as well as long-term impacts on weight management. In addition to selection bias and a lack of true RCT, a challenge with research in this area is defining self-efficacy and having a unified measuring system or assessment tool. Policy to support health behavior interventions is necessary to optimally impact the US obesity crisis. Future physical activity related research should be expanded to include a representative sampling of school age participants across a greater diversity of school-based or socially oriented environment.

## Figures and Tables

**Table 1:** Included articles.

Author	Population	Intervention	Comparison	Outcome	Study design
Annesi 2006 [14]	165 adolescent, children, 70% African American	12 week YMCA program (3 days/week): 2003 treatment group-homework, PA, social support, behavior, health education, nutrition education, self-regulatory skills 2005 treatment group-homework, PA, social support, behavior, health education, nutrition education	YMCA participants, unstructured program	2005 treatment improved self-efficacy	O
Annesi et al. 2007 [16]	392 adolescent, children, African American	12 week YMCA program (3 days/week): homework, tutoring, PA, social support, behavior, health education, nutrition education	Physical education class YMCA charter school, same as intervention	Intervention and control improved self-efficacy	O
Annesi et al. 2008 [15]	269 children, African American, BMI > 95%	12 week YMCA program (3 days/week): homework, snack, PA, nutrition education, health education, behavior, social support	YMCA wait listed group homework and snack	Intervention improved self-efficacy	O
Dishman et al. 2004 [17, 24]	2744 adolescent females, 24 schools	2 year school based program: physical education, health education, social support (frequency not reported)	Standard physical education	No effect of program on self-efficacy	RCT
Jamner et al. 2004 [18]	58 adolescent females, group assignment	4-month school-based program (5 days/week): physical education, social support, health education	Not described	Intervention improved self-efficacy	QE
Lubans et al. 2010 [19]	108 adolescent, Austrian secondary school	8-week school-based program (2 days/week): resistance training during lunch	Usual diet and activity	No statistically significant improvement in self-efficacy	RCT
Stevens et al. 2003 [20]	1447 children, 41 American Indian schools	3-year school-based program: school lunch, physical education, education nutrition exercise, social support (frequency not reported)	Not described	1999 and 2000 improved PA self-efficacy, female only	RCT
Verstraete et al. 2007 [21]	764 children, 16 Belgium schools	2-year school-based program: physical education, health education, PA (frequency not reported)	Not described	No effect of program self-efficacy	RCT
Wilson et al. 2002 [23]	53 adolescents, children, African American 30% ideal weight for height	12-week after-school program (3 days/week): social cognitive theory (SCT) (nutrition education, PA, behavior) or SCT/Motivational Intervention (nutrition education, PA, behavior) videotape interview	Usual diet, PA, health education material	SCT/Motivational Intervention improved PA self-efficacy	QE
Wilson et al. 2005 [22]	48 adolescent, children, 83% African American, underserved area	24-week school-based program (3 days/week): homework, PA, SCT/Motivational Intervention videotape interview	General health education	No effect of program on self-efficacy	QE

Observational study design (O), physical activity (PA), quasiexperimental study design (QE), randomized control trial (RCT).

**Table 2:** Self-efficacy assessments.

Author	Self-efficacy assessment
Annesi 2006 [14], Annesi et al. 2007 [16], Annesi et al. 2008 [15]	Perceived barriers
Dishman et al. 2004 [17, 24]	Self-efficacy
Jamner et al. 2004 [18]	Overcoming internal and external barriers to exercise
Lubans et al. 2010 [19]	Resistance training self-efficacy and outcome expectancy
Stevens et al. 2003 [20]	Diet and exercise self-efficacy
Verstraete et al. 2007 [21]	Perceived barriers and benefits self-efficacy for physical activity
Wilson et al. 2002 [23]	Diet and exercise self-efficacy
Wilson et al. 2005 [22]	Exercise self-efficacy

**Table 3:** Outcome improvements.

Author	Self-efficacy improvement	Changes in body mass
Annesi 2006 [14], Annesi et al. 2007 [16], Annesi et al. 2008 [15]	Yes	Not indicated
Dishman et al. 2004 [17, 24]	Yes	Not indicated
Jamner et al. 2004 [18]	No	Not indicated
Lubans et al. 2010 [19]	No	Positive effect on body composition, not correlated to self-efficacy
Stevens et al. 2003 [20]	Yes, female only	Not indicated
Verstraete et al. 2007 [21]	No	Not indicated
Wilson et al. 2002 [23]	Yes	Not indicated
Wilson et al. 2005 [22]	No	Not indicated

**Table 4:** Oxford/Jadad rating.

Author	Oxford	Jadad
Annesi 2006 [14]	2b	NA
Annesi et al. 2007 [16]	2b	NA
Annesi et al. 2008 [15]	2b	NA
Dishman et al. 2004 [17, 24]	1b	1a
Jamner et al. 2004 [18]	1b	1a
Lubans et al. 2010 [19]	2b	3a, b, c
Stevens et al. 2003 [20]	1b	1a
Verstraete et al. 2007 [21]	1b	2a, c
Wilson et al. 2002 [23]	2b	NA
Wilson et al. 2005 [22]	1b	1a

Oxford designation: individual RCT (1b), individual cohort study (2b).

Jadad designation: (1 point assigned for each a, b, c) randomization mentioned (a), randomization was appropriate (b), the fate of all participants in the study is known (c). Not applicable due to study design (NA).

**Table 5:** Eliminated articles and references.

Article	Population	Study design	Exclusion rationale
Allison et al. [25]	a	O	NCI, SEIE
Annesi et al. [26]	c	O	NC
Annesi et al. [27]	c	O	NC, SEIE
Annesi [28]	c	O	NC
Annesi et al. [29]	c	O	NC
Annesi et al. [30]	c	O	NC
Annesi et al. [31]	c	O	NC
Annesi [7]	a/c	M	SEIE
Barr-Anderson et al. [32]	a	O	NCI, SEIE
Barr-Anderson et al. [33]	a	O	NCI, SEIE
Beets et al. [34]	a	O	NCI
Berry et al. [35]	a	O	NCI
Boutelle et al. [36]	A	O	NCI
Bray [37]	a/A	O	NCI
Brown [38]	A	O	NCI
Cardon et al. [39]	c	O	NC, NCI
Carels et al. [40]	A	RCT	NSE
Courneya and McAuley [41]	A	O	NCI, SEIE
de Bourdeaudhuij et al. [42]	a	O	NCI, SEIE
Deforche et al. [43]	a	O	NC, NCI
Dilorenzo et al. [44]	a/c	O	NCI
Dishman et al. [45]	a/c	O	NCI, SEIE
Dzewaltowski et al. [46]	a	O	NCI, SEIE
Dzewaltowski et al. [47]	c	O	NCI, SEIE
Epstein et al. [48]	c	QE	NCI, NSE
Epstein et al. [49]	a	QE	NSE
Foster et al. [50]	c	O	NCI
Gao et al. [51]	A	O	NCI, SEIE
Gillison et al. [52]	a	O	NCI, NSE
Gortmaker et al. [53]	a	QE	NSE
Hausenblas et al. [54]	a	O	NCI, SEIE
Heitzler et al. [55]	c/A	O	NCI, SEIE
Keats et al. [56]	a	O	NCI
Kitzman-Ulrich et al. [57]	a	O	NC, SEIE
Kloek et al. [58]	A	O	NCI, SEIE
Knöpfli et al. [59]	A	QE	NCI, NSE
Kowal and Fortier [60]	A	O	NCI, NSE
Lewis et al. [8]	c/A	R	SEIE
Lytle et al. [61]	a/c	O	SEIE
Maltby and Day [62]	A	O	NCI, NSE
Martin et al. [63]	a	O	NSE
Martin and McCaughtry [64]	c	O	NCI, SEIE
McClaran [65]	A	O	NC
Melnyk et al. [66]	a	RCT	NSE
Mildestvedt and Meland [67]	A	O	NCI, NSE
Moreno Murcia et al. [68]	a	O	NSE
Moreno et al. [69]	A	O	NCI
Motl et al. [70]	a	QE	NSE
Motl et al. [71]	a	RCT	SEIE
Murru and Ginis [72]	A	RCT	NCI, SEIE
Nicholls et al. [73]	A	O	NCI, SEIE
Nigg and Courneya [74]	a	O	NCI, SEIE
Nigg [75]	a	O	NCI
Parcel et al. [76]	a	QE	Year prior 2000
Patrick et al. [77]	a	QE	NCI, NSE
Pender et al. [78]	a/c	O	NCI
Quintiliani et al. [79]	a/A	O	NCI
Raudsepp et al. [80]	a	O	NSE
Renner et al. [81]	a/A	O	NCI, SEIE
Robbins et al. [82]	c/A	O	NCI
Robbins et al. [83]	a/c	O	NCI, SEIE
Roemmich et al. [84]	a	QE	NSE
Roemmich et al. [85]	a/c	RCT	NSE
Rosenberg et al. [86]	a	RCT	NSE
Ryan et al. [87]	A	O	NCI, NSE
Ryan and Dzewaltowski [88]	a/c	O	NCI, SEIE
Sallis et al. [89]	A	O	NCI
Sallis et al. [90]	a/c	O	NSE
Sallis et al. [9]	a/c	R	SEIE
Salmon et al. [91]	c	RCT	NSE
Salvy et al. [92]	a	QE	NCI, NSE
Schneider et al. [93]	A	RCT	NSE
Sherwood et al. [94]	c	RCT	SEIE
Shields et al. [95]	a	O	NCI, SEIE
Shields and Brawley [96]	A	O	NCI, SEIE
Shrigley and Dawson [97]	A	O	NC
Sothern et al. [98]	c	O	NCI, SEIE
Stone et al. [99]	a/c	R	SEIE
Strauss et al. [100]	a/c	O	NCI
Sung et al. [101]	c	O	NCI, NSE
Taylor et al. [102]	a/c	O	NCI
Taymoori and Lubans [103]	a	QE	NCI, SEIE
Taymoori et al. [104]	a	O	NCI, SEIE
Thompson et al. [105]	c	O	NCI, NSE
Trost et al. [106]	c	O	NCI, SEIE
Trost et al. [107]	a	O	NCI, SEIE
Valois et al. [108]	a	O	NCI
Watson et al. [109]	a/c	O	NC, NCI
Wilson et al. [110]	a	O	NCI, SEIE
Wilson et al. [111]	a	RCT	SEIE
Wenthe et al. [112]	a	O	NCI, SEIE
Wright et al. [113]	a	O	NCI, SEIE

Adults (A), adolescent (a), children (c), literature or systematic review (R), meta-analysis (M), no control group (NC), intervention criteria not fulfilled (NCI), self-efficacy not measured (NSE), observational study design (O) (includes cross-sectional study design, longitudinal design, cohorts, cross-over design), quasiexperimental study design (QE), randomized control trial (RCT), self-efficacy influencing exercise (SEIE).

## Appendix

### Database Search

For the publications, all titles and abstracts were reviewed as per study inclusion/exclusion criteria.

#### A.1. PubMed

The selection of medical subject heading terms (MeSH) was applied toward the PubMed search. Applying “obesity” as a MeSH term to those listed in the following diminished the number of displayed results. Additionally, it did not contribute to the number of potential inclusion articles. The MeSH term “physical activity” displayed results for “motor activity” suggesting a developmental skill rather than exercise.

(1) Exercise and self-efficacy. (2) Exercise influence on self-efficacy. (3) Exercise and motivation. (4) Exercise and behavior.

Limits: ages 0–18 years, clinical trials, reviews, randomized control trials, years 2000–2011.

#### A.2. Web of Knowledge

(1) Exercise and self-efficacy.

Limits: ages 0–18 years, clinical trials, reviews, randomized control trials, years 2000–2011.

#### A.3. PsychInfo

(1) Exercise and self-efficacy.

Limits: ages 0–18 years, clinical trials, reviews, randomized control trials, years 2000–2011.

#### A.4. Cochrane Central Register

(1) Exercise influencing self-efficacy in children and adolescents, included advance search.
